# An Overview of Depression among Transgender Women

**DOI:** 10.1155/2014/394283

**Published:** 2014-03-13

**Authors:** Beth Hoffman

**Affiliations:** Department of Public Health, California State University Los Angeles, Los Angeles, CA 90032, USA

## Abstract

Rates of depression are higher in transgender women than in the general population, warranting an understanding of the variables related to depression in this group. Results of the literature review of depression in transgender women reveal several variables influencing depression, including social support, violence, sex work, and gender identity. The theoretical constructs of minority stress, coping, and identity control theory are explored in terms of how they may predict depression in transgender women. Depression and depressive symptoms have been used to predict high-risk sexual behaviors with mixed results. The implications of the findings on treating depression in transgender women include taking into account the stress of transition and the importance of supportive peers and family. Future studies should explore a model of depression and high-risk behaviors in transgender women.

## 1. Introduction

Transgender is a general term that refers to people with a variety of identities. In the broadest sense it refers to people who do not adhere to the cultural definitions of gender [1]. This term encompasses transsexual men and women, or those that have altered their genitals to match the gender they choose to express, but it also includes transgender men and women who express a gender identity other than the one assigned at birth but do not choose to have genital alteration surgery [2]. Both transgender and transsexual people may use hormones, clothing, makeup, wigs and/or hairstyles, and other types of surgery (such as facial feminization surgery or mastectomies) in their presentation of gender. Transgender also may refer to drag kings and queens, women and men who dress as the opposite gender for the purposes of performance, or people who identify as genderqueer—people who choose to express both or neither gender in their presentation.

Transgender women experience depression, suicidal ideation, and suicide attempts at rates much higher than in the general population: estimates of the lifetime prevalence of depression in transgender women have been reported as high as 62% [3], while the lifetime depression rate for the general United States population is 16.6% [4]. The high rate of depression is not surprising given the discrimination transgender women face. However, it is important to understand the factors influencing depression in this population as well as how depression influences other factors affecting transgender women. Such an understanding will aid in the development of effective modes of treatment for depression in transgender women.

This paper reviews the burgeoning literature on depression in transgender women. The author searched the databases PSYCINFO, MEDLINE, and CINAHL using the keywords transgender and depression to find articles and also culled citations within the articles found for other relevant papers. The majority of research has focused on the factors influencing depression and suicidal ideation (Table 1). Second, theoretical constructs for the high rates of depression in this population are discussed. Finally, three known studies have included depression as a variable that influences health behaviors in this population; findings of these studies are presented. The paper concludes with implications for clinicians working with transgender women.

All articles that included transgender women in the sample pool and discussed relationships between depression and other variables were included. A total of fourteen articles were found and are discussed within. Most of these studies either did not mention how they chose their sample of transgender women [13, 15] or allowed women to self-identify as transgender [2, 3, 5, 9, 10, 12, 16, 17]. Budge and colleagues [11] included only transgender people (men and women) who identified on what they called a “binary gender identity,” in other words, participants who used to identify as one gender and now identified as the other gender. This has the potential of excluding many individuals who consider themselves transgender but are along a spectrum of transition. In a series of studies, Nuttbrock and colleagues [7, 8, 14] used a definition for inclusion that a participant must have been assigned the sex of male at birth but did not consider themselves “completely male” in all areas of life. This allows much more inclusion for people who may be early in transition or for people who are living as a female in only certain social spheres or times.

Study recruitment methods varied, but only two studies [12, 15] were recruited among high-risk groups of sex workers. The other studies recruited via venues that are not likely to yield only high-risk participants such as a conference [9] and online [2, 11]. The majority of the other studies recruited as widely as possible, targeting community groups (some of which do cater to HIV-positive individuals) but also using respondent-driven sampling to reach transgender females who would not be reached otherwise.

## 2. Factors Predicting Depression

Variables that are proposed to predict depression in transgender women include both interpersonal and intrapersonal constructs. The author has discussed variables that do not show a relationship with depression in the sample where the lack of relationship was indicated in the studies.

*Social support* is a measure that examines the availability of people in one's life who provide emotional and mental resources for coping. Often this is broken into categories such as family support and peer (or friend) support. Family social support is often less available for transgender women as families react to the transition and may reject the transitioning family member [15, 18]. Peer support varies, as transition might lead to rejection by a women's pretransition peer group but transition often leads to acceptance by other transgender women, particularly in urban areas [19].

Social support does seem to reduce the risk of depression in transgender women. Several studies have found a direct relationship of family and peer support associated with better mental health [2, 11, 15]. Nemoto et al. [12] suggest that it is not just the presence of social support in the past month (from family and both transgender and nontransgender friends) but rather the satisfaction with this support that is protective against depression.

Though it is not conclusive, peer support may not be as important as family support in predicting depression [2]. However, this study measured family and peer support differently; family support was assessed via items regarding perceived supportiveness of parents, siblings, partners, and children while peer support was assessed via measures of the proportion of time spent with other transgender people and perceived feelings of isolation in being transgender. The inequity of these measures is problematic in terms of comparing these measures. Another study suggests that the influence of social support, at least superficially in terms of accepting versus rejecting the transgender woman's gender identity, may vary based on life stage. More specifically, acceptance/rejection by parents and siblings matters until middle age, when acceptance/rejection by a sexual partner matters from young adulthood [14].

*Violence.* There are three categories of violence often discussed in the literature of transgender women: physical violence, sexual violence, and verbal harassment (sometimes labeled as stigma or discrimination). Transgender women are at risk of violence, including physical violence and discrimination, at rates between 2 and 3 times those of people who are not transgender women [20]. Violence against transgender women can damage the woman's sense of self and being, further intensifying the damage [8]. Studies that examined effects of physical and verbal violence in aggregate indicated that violence against transgender women is associated with increased depression [8] and may be a predictor of depression [7].

Physical and verbal abuse may have different impacts on victims than sexual abuse. Testa and colleagues [10] found that physical violence was associated with suicidal ideation and attempts, while sexual violence was associated with increased substance use. A study examining sexual violence found an association between sexual partner violence and depression [5], indicating that the relationship between the transgender woman and the perpetrator of the violence may be an important factor. Nemoto et al. [12] found that despite high rates of physical and sexual violence (half of participants reported victimization), only verbal violence predicted depression in a multiple regression model. Bockting and colleagues [2] found similar results. These results contrast with an earlier study that found that physical and sexual violence, but not verbal violence, predicted attempted suicide [6]. Perhaps the dynamics behind depression are different than the ones behind suicidal attempts.

*Sex Work.* Many transgender women engage in sex work. Often it is the only viable source of income due to workplace discrimination and it is certainly the most lucrative form of income for transgender women who need to finance expensive surgeries and/or hormone treatments [21, 22]. Sex work is very common in this population: as many as 67% of transgender women between 15 and 24 have engaged in sex work at some point in their lives [23].

The relationship, if any, between sex work and depression is unclear. Several studies show no relationship between sex work and depression [5, 7, 12]. Yet several factors leading to depression, such as a lack of social support and a lower education status, are associated with engagement in sex work as well [23]. In addition, transgender sex workers have stated during interviews and focus group-based studies that they engage in drug use to escape the stress of sex work [21, 24], indicating that mental distress is indeed occurring. Though interviews with transgender sex workers indicate that high rates of drug use among transgender women are due to an attempt to deal with mental stress and depression [21], no quantitative studies to date have examined whether drug use is associated with depression in transgender women.

*Gender Identity.* The very term transgender implies movement, a crossing (trans) of gender identities. This crossing is commonly called transition and takes place over a span of time that may last for several years. The process is affected by the social, cultural, developmental, and individual factors and economic and social resources of the person transitioning. Part of this transition entails resolving issues of conflict regarding gender identity, often by altering one's behavior and presentation to better fit a gender identity that did not mesh with one's gender at birth [15].

Gender identity is a complex, multidimensional concept and researchers have conceptualized it in many ways. Bockting and colleagues [2] examined several aspects of gender identity, including gender dysphoria, investment in being perceived as their gender of expression, and openness and pride about being transgender. Of these, only transgender pride and openness (feeling that being transgender made her special, and that she was comfortable revealing that she was transgender to others) was negatively associated with mental distress. In a study of the association between feelings about being transgender and psychological distress (anxiety, depression, and somatization), Sánchez and Vilain [9] found that fear about how being transgender would affect life was associated with psychological distress, but other thoughts and feelings about being transgender were not predictive of distress. Budge et al. [11] examined the feelings of loss a transgender person has regarding aspects of life that have deteriorated since transitioning, including job, housing, family relations, friendship, and finances. Though this variable was not predictive of depression in their final structural equation model, they did find that transgender women experienced more loss than did transgender men, indicating that transgender women may experience more discrimination than do their male counterparts.

*Sociodemographic Variables.* Most studies included demographic variables that moderated the main variables of study. Many of the results assessing these variables are mixed. Age is a common demographic control; several studies indicate that being younger is associated with more depression [2, 6, 7], while still other studies found no association between depression and age [8, 12]. A younger transgender woman may not have developed the capacities to cope with the stressors of life as transgender or may be more entrenched in the stressful transition process, leading to greater depression among younger women in some studies. The majority of studies assessing it found no association between race/ethnicity and depressive symptoms [6, 12] but Nuttbrock, and colleagues [8] found that Black transgender women had lower rates of clinical depression than did other racial/ethnic groups, indicating that depressive symptoms and clinical depression may have different correlates.

Less education predicted more depression in two studies [2, 5] but was not correlated in a third study [6]. At first glance education is a proxy for income, but the relationship between education and income does not only hold for transgender populations due to widespread discrimination. Indeed, income appears to be unrelated to depression [2, 5, 11]. However, unemployment was a risk factor for depression in a sample of transgender women in Ontario, Canada [13]; perhaps a lack of steady income is a stressor regardless of the amount one's income is in general.

## 3. Theoretical Constructs Explaining Transgender Women's Depression

Researchers studying depression in transgender women have utilized several sociopsychological theories to account for the choices of variables to study. These constructs are presented next, roughly in order from the most to the least researched as of this point.

*Minority Stress.* This concept is derived from research literature into mental health of lesbians and gay men [25, 26], which in turn was based on similar theories for other minority groups. In our context, the definition of minority stress is that the discrimination and stigmatization transgender people face lead to stress, which in turn leads to depression and other negative mental health outcomes. It seems clear that discrimination, both actual in the form of verbal, physical, and/or sexual violence and perceived stigma, does lead to depression and suicide attempts [2, 5–7, 9, 10]. Conversely, positive feelings about being transgender are associated with less depression [9].

Transgender discrimination differs from other forms of discrimination in several ways. Transgender women may find themselves facing rejection from the family of origin due to their transition [15, 18], while a person of a given race/ethnicity will continue to have acceptance from their family on the basis of homogeneity alone. Second, though exact numbers are unknown for several reasons (including reluctance of transgender people to report being victims of violence and a lack of identification of transgender status in police data), transgender women face high rates of violent discrimination, leading to assault, sexual violence, and murder [27].

*Coping* is the process by which people use various psychological and behavioral mechanisms to manage reactions to stress. Coping mechanisms are generally divided into two categories: avoidant and facilitative coping. Avoidant coping is considered an unhealthy way of dealing with stress, as the person minimizes or avoids thinking about the problem via cognition, distraction, overeating, or substance use, which can lead to more stress or additional health problems. Facilitative coping, or addressing the problem via support of others, learning new skills, or changing behavior is considered healthier, as the problem is resolved in a positive manner via social support, changing behavior, or learning new skills [9, 11, 28]. Greater use of facilitative coping would therefore be predicted to be protective against depression while avoidant coping would place one at risk of depression.

Studies of transgender women have indicated that approaches indicative of avoidant coping, such as substance use, were predictive of depression [6], while facilitative coping mechanisms like utilizing social support led to less depression [11, 13, 15]. Avoidant and facilitative coping may interact in terms of their role in predicting depression, as Budge and others [11] found that avoidant coping mediates the relationship between social support and depression. In addition, depressed individuals may reject social support, further deepening their negative mood state [12]. While these studies do lend support to a model of avoidant and facilitative coping affecting mental effect, most studies are examining only a small sample of the variations in each type of coping. Avoidant coping is more than substance use and facilitative coping is more than calling on social support. To date no studies have examined the effects of avoidant and facilitative coping in totality.

*Identity control theory* originated in social psychology and centers on the balance between a person's identity and the labels and expectations of others in the person's life. According to this theory, a person compares her identity against the perceptions of her from others in her life. If there is a discrepancy between the identity and the perceptions then the person changes her behavior to bring these into alignment [29]. A transgender woman would be balancing her identity as female with the perceived attitudes of friends and family (among others), which would be expressed as rejection or support of her as a transgender woman. Rejection of her identity would be associated with greater risk of depression.

Only one research team to date has examined identity control theory in the context of depression among transgender women. Following a preliminary study indicating that both friend and family supports were protective against depression [15], Nuttbrock and colleagues [14] examined conflict and affirmation reactions to disclosures of gender identity among parents, siblings, classmates, friends, coworkers, and sex partners of transgender women and whether these reactions influenced depression. They found that conflict was a risk factor for depression and affirmation was protective against depression, supporting identity control theory and indicating the importance of a transgender woman's social network in her mental health.

## 4. Depression as a Factor Predicting Health-Related Variables

Due to extremely high rates of HIV among transgender women (approximately 27% of transgender women are HIV positive, according to Herbst et al. [30]), researchers have examined factors contributing to higher risk of HIV or of behaviors that put one at risk for HIV, such as unprotected receptive anal intercourse (URAI). Three known studies have included depression as a predicting variable (Table 2). It is unclear whether depression contributes to HIV, as Garofalo et al. [16] found no association between depression and HIV while Nuttbrock et al. [7] found that depressive symptoms increased the risk of contracting HIV or other sexually transmitted infections. Similarly, results indicating a relationship between URAI and depression are also mixed, with Nuttbrock et al. [7] reporting a positive association between URAI and depression and Operario and Nemoto [17] reporting no association (though they did report an association between suicide attempts and URAI).

## 5. Conclusions and Implications

Though the literature regarding depression in transgender women is still sparse, a few key findings do emerge. It is clear that interactions with other people, whether positive in terms of social support or negative in terms of rejection or violence, are important factors predicting depression in transgender women. Second, the very process of transitioning is stressful and this stress may contribute to depression in transgender women. Results regarding gender identity, social support across the lifespan, and identity control all support this assumption. Finally, the relationship between sex work and depression is not clearly understood. Given the relationship of sex work to other risk behaviors which lead to higher risk of HIV [19], and given the pervasive workplace discrimination suffered by transgender women which contributes to engagement in sex work, it is important to continue research into how sex work affects the mental (as well as physical) health of transgender women.

The variables surrounding a transgender woman's physical and emotional health are complex. To date nobody has tested a model that attempts to describe the dynamic relationship between sexual risk behaviors, transgender identity and support, and mental health, though the author has proposed a preliminary model [31]. Still other studies are needed to fully elucidate the many relationships between depression and predictor variables in the population.

Though these studies are ostensibly about depression in transgender women, only two of these studies [8, 14] measured clinical depression among study populations. The other studies assessed prevalence of depressive symptoms, which may not be measuring clinical depression. As suggested by the different associations for Black transgender women for clinical depression, studies indicating associations of variables with depressive symptoms may not mean that clinical depression will be associated with these variables.

Of the fourteen studies described here, eleven were cross sectional, which limits the conclusions that can be drawn regarding factors that precede depression because of both the lack of time as a factor and the possibility that third variables may be driving the variable relationships seen. The studies examining participants longitudinally [7, 8, 14] are important additions to the body of knowledge, as transitioning is a dynamic process that almost necessitates the inclusion of time when studying health within transgender populations. Future studies should consider a longitudinal study design while addressing the interaction of inter- and intrapersonal variables influencing depression in transgender women. Such studies will elucidate the variables affecting health in transgender women, providing indications to be used in creating effective interventions to reduce depression, drug use, and HIV among this population.

## Figures and Tables

**Table 1:** Factors predicting depression-related variables (by theory).

Authors, year of publication	Study location	Study population	Study method	Predicted variable	Correlates tested	Control variables	Outcomes
Minority stress model							

Bazargan and Galvan (2012) [5]	Los Angeles	220 MTF low-income Latina women	(i) Interview (ii) Cross sectional	Depression	(i)** Verbal violence** (ii)** Sexual partner violence** (iii) History of sex work	(i) **Education** (ii) Living arrangement (iii) Income (iv) Immigration status (v) Years in US	Verbal violence, sex partner violence, and ed. of ≤11th gr.→depression.

Bockting et al., (2013) [2]	United States	629 MTF, 464 FTM, recruited online	(i) Online survey (ii) Cross sectional	Mental health (anxiety and depression)	(i)** Verbal violence** (ii)** Perceived stigma** (iii) Gender dysphoria (iv) Investment in passing (v)** Outness** (vi)** Family support** (vii) Peer support	(i) **Age** (ii) **Education** (iii) Income (iv) Marital status (v) Urban versus rural	Verbal violence, perceived stigma, family support, outness, younger age, and less education→worse mental health.

Clements-Nolle et al., (2006) [6]	San Francisco	392 MTF, 123 FTM, respondent-driven sampling	(i) Interview (ii) Cross sectional	Att. suicide	(i)** Depression** (ii) Self-esteem (iii)** Substance use** (iv) **Discrimination** (v) Verbal violence (vi)** Physical violence** (vii)** Sexual violence**	(i) Race/ethnicity (ii) **Age** (iii) Sexual orientation (iv) Education (v) Recent employment (vi) Incarceration (vii) Know HIV status	Younger age, depression, alcohol and drug treatment, rape, and phys. gender discrimination→suicide attempt.

Nuttbrock et al., (2013) [7]	New York City	230 MTF HIV negative at baseline, recruited throughout community	(i) Interview (ii) 3-year prospective	Depr. symptoms	(i)** Physical violence** (ii)** Verbal violence**	**Age**	Violence→depr. symptoms.

Nuttbrock et al., (2010) [8]	New York City	571 MTF, recruited throughout community	(i) Interview (ii) Retrospective longitudinal	Major depr., suicidality	(i)** Verbal violence** (ii)** Physical violence** (iii) Use of hormones (iv) “Coming out” to friends/family	(i)** Episodic or persistence of violence** (ii)** Life stage** (iii) Age (iv)** Ethnicity**	(i) Victims of violence 2x as likely depr., suicidal (ii) Persist. psych. abuse→depr., suicidality in adol. (iii) Period. and persist. phys. abuse→depr. and suicidality (iv) Blacks ↓rates of depression

Sánchez and Vilain (2009) [9]	Arizona and California	53 MTF, recruited at conferences	Paper survey	Psychological distress (somatization, depression, anxiety)	Collective self-esteem		Fear about identity→greater psyc. distress

Testa et al., (2012) [10]	Virginia	350 adult students (179 MTF, 92 FTM), recruited via service providers, support groups, and peer networks	Survey: Paper (English and Spanish), Internet (English) Cross sectional	(i) Suicidal ideation, attempts (ii) Substance abuse	(i)** Physical violence** (ii)** Sexual violence**		(i) Phys. violence→↑suicidal ideation attempts (ii) Sex violence ↑substance abuse

Coping							

Budge et al., (2013) [11]	United States	226 MTF, 125 FTM, recruited online	(i) Online survey (ii) Cross sectional	Depression Anxiety	(i)** Social support** (ii) Facilitative coping (iii) Avoidant coping (iv) Transspecific loss	(i) Transition status (ii)** Family history** (iii) Age Income	Family history and SS→depression. Transition status neg.→av. coping, av. coping mediates SS and depression.

Nemoto et al., (2011) [12]	San Francisco and Oakland	573 MTF w/sex work history	Interview Cross sectional	Depression	(i) Physical violence (ii)** Verbal violence** (iii)** Social support** (iv)** Suicidal ideation**	(i) Ethnicity (ii) Age (iii) Immigration (iv) **Education** (v)** Income** (vi) Sex work (vii) Housing status (viii) Sexual orientation (ix) SRS	Depression→higher need for, less reception of, and dissatisfaction with social support, transphobia, suicidal ideation, lower income and education.

Rotondi et al., (2011) [13]	Ontario	186 MTF, respondent-driven sampling	Online and paper survey	Depression		(i) Age (ii) Relationship status (iii)** Rural versus urban** (iv) Income (v)** Employment** (vi) Education (vii) Housing (viii) **Childhood abuse** (ix) Transphobia (x) Racism (xi) Health probs. (xii)** Social support** (xiii) Transition (xiv)** Passing** (xv) Living as gender	(i) Unemployment, childhood abuse, living in rural Ontario→↑risk of depression. (ii) Support, passing rarely or often (not always) and little community org. involvement→↓risk of depression

Identity control theory							

Nuttbrock et al., (2011) [14]	New York City	571 MTF, recruited throughout community	(i) Interview (ii) Retrospective longitudinal	Major depr.	**Gender identity conflict/affirmation **	(i)** Relationship type** (ii)** Life stage**	Parents, siblings, fellow students c/a→depression until middle age, sex partner c/a→from young adulthood on.

Nuttbrock et al., (2002) [15]	New York City	43 MTF sex workers	(i) Not specified (ii) Cross sectional	Depr. symp.	(i)** Family support** (ii)** Friend support**		Neg.→between depr. symptoms and family/friend support for trans. identity

**Table 2:** Depression as a factor predicting health-related variables.

Authors, year of publication	Study location	Study population	Study method	Predicted variable	Correlates tested	Outcomes
Garofalo et al., (2006) [16]	Chicago	51 MTF minorities, 16–25 years (HIV funding, community agency)	Survey Cross-sectional	HIV	Social support Life stress Sexual risk cognition Self-esteem Depression Risk behaviors	Depression rates similar to general population. No variables assoc. w/HIV status except African-American.

Nuttbrock et al., (2013) [7]	New York City	230 MTF, HIV negative at baseline, recruited throughout community	Interview 3-year prospective	HIV STDs **Unprotected receptive anal intercourse (URAI)**	**Depr. symptoms**	Depr. symptoms led to higher risk of URAI. Depr. symptoms increased risks of HIV/STIs.

Operario and Nemoto, 2005 [17]	San Francisco	110 API MTF, venue based recruiting (and AIDS service orgs.)	Interview	URAI	**Suicide attempt** Depressed **Sex work** Sex assign. surgery HIV status	Sex work in past month and ever attempted suicide associated with URAI.

## References

[1] Bockting WO (1999). From construction to context: gender through the eyes of the transgendered. *SIECUS Report*.

[2] Bockting WO, Miner MH, Romine RES, Hamilton A, Coleman E (2013). Stigma, mental health, and resilience in an online sample of the US transgender population. *The American Journal of Public Health*.

[3] Clements-Nolle K, Marx R, Guzman R, Katz M (2001). HIV prevalence, risk behaviors, health care use, and mental health status of transgender persons: implications for public health intervention. *The American Journal of Public Health*.

[4] Kessler RC, Berglund P, Demler O, Jin R, Merikangas KR, Walters EE (2005). Lifetime prevalence and age-of-onset distributions of DSM-IV disorders in the national comorbidity survey replication. *Archives of General Psychiatry*.

[5] Bazargan M, Galvan F (2012). Perceived discrimination and depression among low-income Latina male-to-female transgender women. *BMC Public Health*.

[6] Clements-Nolle K, Marx R, Katz M (2006). Attempted suicide among transgender persons: the influence of gender-based discrimination and victimization. *Journal of Homosexuality*.

[7] Nuttbrock L, Bockting W, Rosenblum A (2013). Gender abuse, depressive symptoms, and HIV and other seuxally transmitted infections among male-to-female transgender persons: a three-year prospective study. *The American Journal of Public Health*.

[8] Nuttbrock L, Hwahng S, Bockting W (2010). Psychiatric impact of gender-related abuse across the life course of male-to-female transgender persons. *The Journal of Sex Research*.

[9] Sánchez FJ, Vilain E (2009). Collective self-esteem as a coping resource for male-to-female transsexuals. *Journal of Counseling Psychology*.

[10] Testa RJ, Sciacca LM, Wang F (2012). Effects of violence on transgender people. *Professional Psychology Research and Practice*.

[11] Budge SL, Adelson JL, Howard KAS (2013). Anxiety and depression in transgender individuals: the roles of transition status, loss, social support, and coping. *Journal of Counseling and Clinical Psychology*.

[12] Nemoto T, Bödeker B, Iwamoto M (2011). Social support, exposure to violence and transphobia, and correlates of depression among male-to-female transgender women with a history of sex work. *The American Journal of Public Health*.

[13] Rotondi N, Bauer G, Travers R, Travers A, Scanlon K, Kaay M (2011). Depression in male-to-female transgender ontarians: results from the trans PULSE project. *Canadian Journal of Community Mental Health*.

[14] Nuttbrock L, Bockting W, Rosenblum A, Mason M, Macri M, Becker J (2011). Gender identity conflict/affirmation and major depression across the life course of transgender women. *The International Journal of Transgenderism*.

[15] Nuttbrock L, Rosenblum A, Blumenstein R (2002). Transgender identity affirmation and mental health. *The International Journal of Transgenderism*.

[16] Garofalo R, Deleon J, Osmer E, Doll M, Harper GW (2006). Overlooked, misunderstood and at-risk: exploring the lives and HIV risk of ethnic minority male-to-female transgender youth. *Journal of Adolescent Health*.

[17] Operario D, Nemoto T (2005). Sexual risk behavior and substance use among a sample of Asian pacific islander transgendered women. *AIDS Education and Prevention*.

[18] Barrington C, Wejnert C, Guardado ME, Nieto AI, Bailey GP (2012). Social network characteristics and HIV vulnerability among transgender persons in San Salvador: identifying opportunities for HIV prevention strategies. *AIDS and Behavior*.

[19] Sausa LA, Keatley J, Operario D (2007). Perceived risks and benefits of sex work among transgender women of color in San Francisco. *Archives of Sexual Behavior*.

[20] Chestnut S, Dixon E, Jindasurat C (2013). 2012 report on lesbian, gay, bisexual, transgender, queer, and HIV-affected hate violence.

[21] Bith-Melander P, Sheoran B, Sheth L (2010). Understanding sociocultural and psychological factors affecting transgender people of color in San Francisco. *Journal of the Association of Nurses in AIDS Care*.

[22] Clements K, Wilkinson W, Kitano K, Marx R (1999). HIV prevention and health service needs of the transgender community in San Francisco. *The International Journal of Transgenderism*.

[23] Wilson EC, Garofalo R, Harris RD (2009). Transgender female youth and sex work: HIV risk and a comparison of life factors related to engagement in sex work. *AIDS and Behavior*.

[24] Nemoto T, Operario D, Keatley J, Villegas D (2004). Social context of HIV risk behaviours among male-to-female transgenders of colour. *AIDS Care*.

[25] Brooks VR (1981). *Minority Stress in Lesbian Women*.

[26] Meyer IH (1995). Minority stress and mental health in gay men. *Journal of Health and Social Behavior*.

[27] Stotzer RL (2009). Violence against transgender people: a review of United States data. *Aggression and Violent Behavior*.

[28] Lazarus RS, Folkman S (1984). *Stress, Appraisal, and Coping*.

[29] Burke PJ, Ritzer G (2007). Identity control theory. *The Blackwell Encyclopedia of Sociology*.

[30] Herbst JH, Jacobs ED, Finlayson TJ, McKleroy VS, Neumann MS, Crepaz N (2008). Estimating HIV prevalence and risk behaviors of transgender persons in the United States: a systematic review. *AIDS and Behavior*.

[31] Hoffman B (2014). The interaction of drug use, sex work, and HIV among transgender women. *Substance Use and Misuse*.

